# Connexins in the Central Nervous System: Physiological Traits and Neuroprotective Targets

**DOI:** 10.3389/fphys.2017.01060

**Published:** 2017-12-18

**Authors:** Nunzio Vicario, Agata Zappalà, Giovanna Calabrese, Rosario Gulino, Carmela Parenti, Massimo Gulisano, Rosalba Parenti

**Affiliations:** ^1^Section of Physiology, Department of Biomedical and Biotechnological Sciences, University of Catania, Catania, Italy; ^2^Department of Drug Sciences, University of Catania, Catania, Italy

**Keywords:** gap junction, hemichannel, connexin, neurodegeneration, neuroprotection

## Abstract

Cell-to-cell interaction and cell-to-extracellular environment communication are emerging as new therapeutic targets in neurodegenerative disorders. Dynamic expression of connexins leads to distinctive hemichannels and gap junctions, characterized by cell-specific conduction, exchange of stimuli or metabolites, and particular channel functions. Herein, we briefly reviewed classical physiological traits and functions of connexins, hemichannels, and gap junctions, in order to discuss the controversial role of these proteins and their mediated interactions during neuroprotection, with a particular focus on Cx43-based channels. We pointed out the contribution of connexins in neural cells populations during neurodegenerative processes to explore potential neuroprotective therapeutic applications.

## Introduction

Gap junctions (GJs) are pivotal for the development and maintenance of physiological arrangement of multicellular organisms (Kandler and Katz, 1998; Krüger et al., 2000; Roerig and Feller, 2000), playing fundamental roles in a wide range of cellular activities, including cell signaling, differentiation, and growth (Goodenough et al., 1996). These structures act as molecular substrate of intercellular communication constituting so called plaques at sites of cell-to-cell interface but also mediating GJs-independent signaling (Jiang and Gu, 2005; Zhou and Jiang, 2014). In fact, connexins (Cxs), which represent the core proteins of GJs, also organize free hemichannels (HCs) throughout the plasma membrane, allowing complex chemical trafficking between cytoplasm and the extracellular environment (Cherian et al., 2005; Spray et al., 2006).

Disruption of GJs, HCs, and Cxs balance, affecting the finely regulated expression in healthy tissues, allows cell elusion from normal physiological behavior by driving them to pathological conditions with different degrees of severity, including cancer and degenerative processes (Decrock et al., 2015b; Belousov et al., 2017). As such, Cxs expression in tissues and organs from embryo to adult throughout life is strictly regulated. This control is particularly emphasized during the developmental process, in which Cxs levels alterations lead to profound impairment of tissue functions up to lethal phenotypes (Bruzzone et al., 1996; Davies et al., 1996).

In particular, Cxs, GJs, and HCs in the central nervous system (CNS) have always been in the spotlight of research about homeostatic glia/neuron activities as well as aberrant organization in different neurological disorders (Parenti et al., 2010; Orellana et al., 2014; Li et al., 2015; Belousov et al., 2017). In the past years, much interest has been placed on neuroprotective and self-repair processes in the CNS as a tool to approach neurodegenerative disorders. However, the molecular mechanisms underpinning the neuroprotective and regenerative processes are far to be fully elucidated and the exploitation of such a promising approach still remains elusive. In this field, GJs- and HCs-based signaling is one of the most controversial mechanisms that take place during degenerative and repairing processes (Andrade-Rozental et al., 2000). Research focused on these pathways, which takes advantages from pharmacological modulators, gene editing and emerging high resolution imaging techniques, represents an intriguing effort among all the explored neuroprotective strategies in both *in vitro* and *in vivo* experimental models (Beyer and Berthoud, 2002; Wong et al., 2016).

## Structural properties and functions in the central nervous system (CNS)

**Figure 1:**
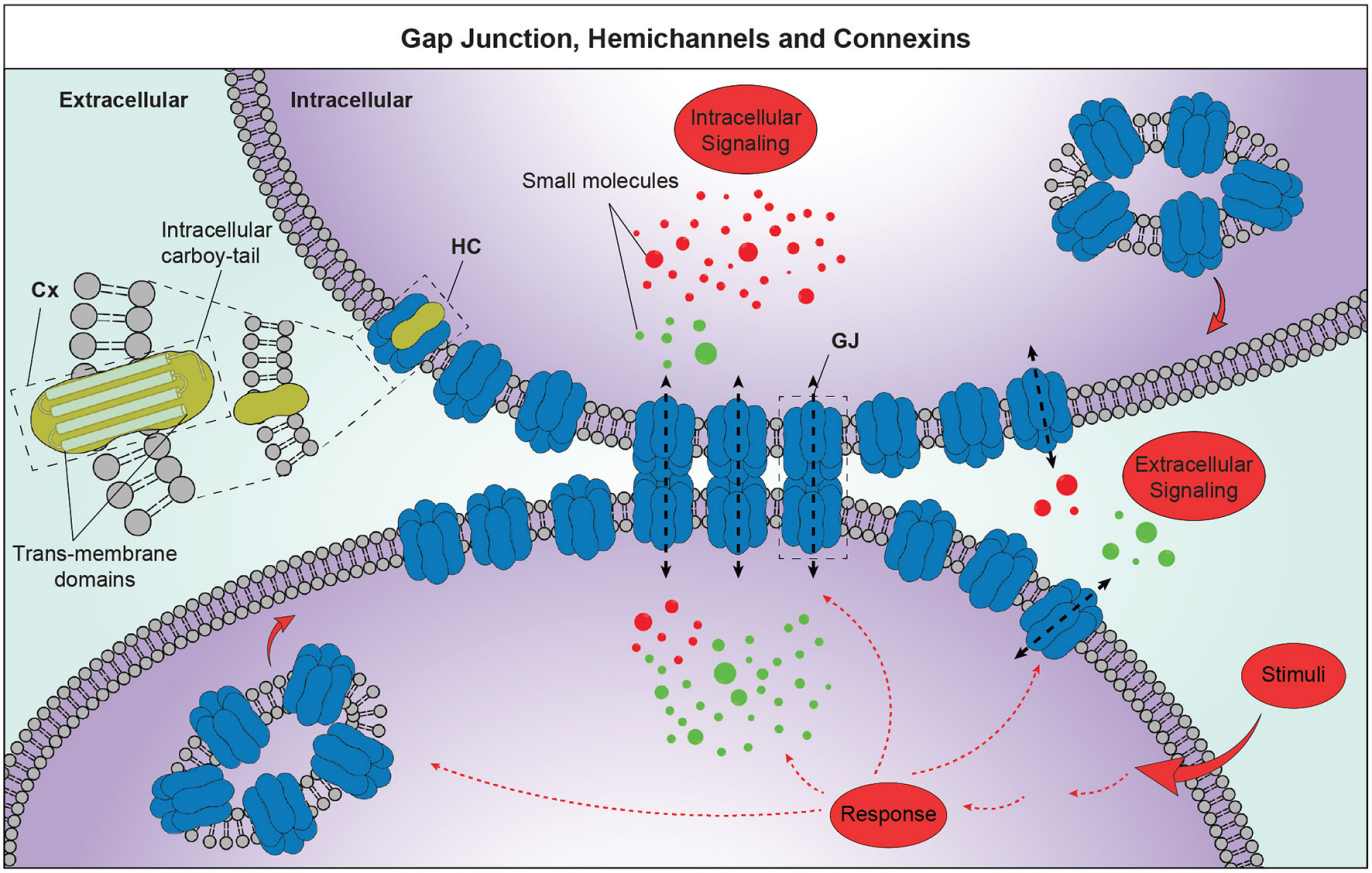
Schematic representation of GJ intercellular communication (GJIC) and HC-mediated cell-to-extracellular environment communication. Cxs, composed by 4 transmembrane domains and an intracellular carboy-tail, are organized to homomeric or heteromeric HCs. GJ plaques are structures of hundreds up to thousands of single channels, which mediate exchanges of small molecules, substrates and metabolites. Those structures show free HCs exposed to the plaque border, where each cell adds newly synthetized HCs. These structures are crucial players of the GJIC and HCs-mediated cell-to-extracellular environment communication and lead to the information exchanges between neighboring cells favoring synchronized and concerted responses. Cx, connexin; HC, hemichannel; GJ, gap junction.

Cxs are encoded by 21 genes in human, each one named according to its theoretical molecular mass in kDa (Willecke et al., 2002). They are structural transmembrane proteins composing HCs, also named connexons, which dock plasma membranes of adjacent cells forming GJs (Bruzzone et al., 1996; White and Bruzzone, 1996). GJs aggregate in specific plasma membrane regions of adjacent cells forming GJ plaques, whichare dynamic macrostructures easily assembled, disassembled, or remodeled configuring a very eventful scenario. In physiological conditions, new HCs are constantly added to the periphery of existing plaques and remain in an inactive conformation until they are aligned with HCs of adjacent cells, while old HCs are removed from the central portion to be destroyed (Gaietta et al., 2002; Figure 1). Finally, Cxs have a few hours half-life, kinetics that are particularly short compared to other plasma membrane proteins (Laird et al., 1991; Lampe, 1994; Beardslee et al., 1998).

Most functions of Cx-formed structures depend on Cxs dynamicity, including different Cxs combinations that convey specific permeability properties and features. In fact, Cxs subunits shape channel conductance, modulate electrical communication and control metabolic coupling between cells (White and Bruzzone, 1996; Salas et al., 2015; Karagiannis et al., 2016). Notably, it is crucial to take into consideration Cxs direct and indirect interactions, which affect many physio-pathological functions (Bruzzone et al., 1996; Cina et al., 2009; Zappalà et al., 2010; Saidi Brikci-Nigassa et al., 2012). On this regard the cytoplasmic tail of Cxs, plays a prominent dynamic role showing different phosphorylation sites and *loci* dedicated to the interaction with other cytoplasmic proteins, modifying the activity of the whole channel (Matsuuchi and Naus, 2013; Kotini and Mayor, 2015).

GJs, HCs, and Cxs play crucial roles in CNS throughout life for several physiological processes being anatomical substrates for electrical and metabolic synchronism. Their importance is evident from the early stages of development, when GJs intercellular communication (GJIC) and cell-to-extracellular environment communications are key events to establish connections, compartmentalization, differentiation, and finally, cell identity (Davies et al., 1996; Bittman et al., 2002; Cina et al., 2007). Even if during adult life some fully differentiated cells do not express high Cxs levels, including some neurons in addition to mature skeletal muscle fibers, red blood cells, and spermatozoids (Bruzzone et al., 1996; Willecke et al., 2002), electrical and metabolic intercellular through GJ- and HC-based coupling remain fundamental in CNS of the adult phenotype (Perlman and Ammermüller, 1994). Cxs also play channel-independent role in cell adhesion, migration, formation of neuronal networks, cellular division, differentiation, and tumorigenicity, acting also synergistically with membranous tunneling tubes (Rimkute et al., 2016). In particular, cell adhesion and migration are key functions during CNS development early in embryonic neuroepithelium and neural migration in neocortex by providing contact interfaces with radial glia (Elias et al., 2007) or along the rostral migratory route of subventricular zone-derived cells (Marins et al., 2009). Cell adhesion is further maintained for astrocytic network stabilization in mature CNS (Haubrich et al., 1996; Lin et al., 2002). Here, complex levels of Cxs organization create a functional unit, named neuro-glio-vascular unit, maintaining both direct cell–cell coupling, via GJIC and paracrine communication via the extracellular compartment properties (Decrock et al., 2015a; De Bock et al., 2017).

A large number of experimental models of human diseases have revealed key Cxs functions in physio-pathological conditions, showing cell type specificity, mutual assistance and redundant role depending on the functional context in which Cxs operate (Nishii et al., 2014). In this field, research has grown and changed remarkably, starting with the discovery of new members of Cx family, describing their spatio-temporal distribution, analysing their functional role and the pathological consequences of their malfunction. In particular, in the neural lineages, Cxs ensure functions ranging from cell division to learning and memory and their disregulation, directly or indirectly conducts to many pathological conditions including epilepsy (Thompson et al., 2008), neuroinflammation (Orellana et al., 2011a; Bennett et al., 2012), neurodegeneration (Orellana et al., 2011b), ischemia (Contreras et al., 2004; Orellana et al., 2010), behavioral alterations (Wang and Belousov, 2011; Zlomuzica et al., 2012; Beheshti et al., 2017) and diverse pathological conditions, including excitotoxic cell-death (Kondo et al., 2000) and injurious depolarization (Schulz et al., 2015; Lapato and Tiwari-Woodruff, 2017).

Several approaches, aiming to modulate channel activity including phosphorylation/de-phosphorylation and nitrosylation until to knockout/knockin technology as well as pharmacological approaches, have come to support their role as emerging therapeutic target in neurodegenerative disorders (Schultz et al., 2016). Thus, by now far from the idea that GJs are simply direct connection between the cytoplasm of two cells, is becoming clear over time that GJs as well as HCs play homeostatic physiological functions whose delicate balance can be altered by leading to pathological conditions of different entities (Table 1).

**Table 1:** Connexins expression and main functions in neurodegeneration.

**Cell type**	**Cxs**	**Gene**	**Ranking**	**Functions**	**References**
Neurons	Cx36	*Gjd2*	+++++	Memory and behavior	Condorelli et al., 1998; Cicirata et al., 2000; Gulisano et al., 2000; Parenti et al., 2000; Bittman et al., 2002; Wang and Belousov, 2011; Zlomuzica et al., 2012; Beheshti et al., 2017
	Cx45	*Gjc1*	+	Memory and behavior	Leung et al., 2002; Cina et al., 2007; Beheshti et al., 2017
	Cx50	*Gja8*	++++	Voltage dependent hemichannel	Beahm and Hall, 2002
Astrocytes	Cx26	*Gjb2*	+++	Degeneration and neurotoxic signaling	Elias et al., 2007; Takeuchi et al., 2011; Koulakoff et al., 2012; Karagiannis et al., 2016
	Cx30	*Gjb6*	++		
	Cx43	*Gja1*	+++++	Adhesion, energy metabolism, and degeneration	Lin et al., 1998; Elias et al., 2007; Pellerin et al., 2007; Takeuchi et al., 2011; Salmina et al., 2014; Suzuki et al., 2014; Salas et al., 2015; Almad et al., 2016
Oligodendrocytes	Cx29	*Gjc3*	+++	Remyelination and regeneration	Altevogt et al., 2002; Nagy et al., 2003a,b; Parenti et al., 2010; Markoullis et al., 2012
	Cx32	*Gjb1*	+++++		
	Cx47	*Gjc2*	++		
Microglia	Cx32	*Gjb1*	+++++	Inflammation	Takeuchi et al., 2006, 2008
	Cx36	*Gjd2*	++	Neurotoxic signaling	Yawata et al., 2008
	Cx43	*Gja1*	+	Inflammation	Orellana et al., 2009
Endothelial cells	Cx37	*Gja4*	+++	Regeneration and healing	Li et al., 2016
	Cx40	*Gja5*	+++++		
	Cx43	*Gja1*	+++++		

*Ranking: +, very low; ++, low; +++, medium; ++++, high; +++++, very high. This table includes information from more than one experimental approach*.

## GJs, HCs, and Cxs: role in neurodegeneration and neuroprotection

Neurodegenerative diseases are among the leading causes of death and disability worldwide. This has led to a growing in-depth research focusing on cellular and molecular mechanisms underlying neurodegeneration to increasingly counteract this phenomenon. In human and in experimental models, a number of Cx alterations are differently involved in the development of various neurodegenerative diseases so much so that they are considered important therapeutic targets (Belousov et al., 2017; Charvériat et al., 2017; Liu et al., 2017). Several independent studies have pointed out that onset and progression of homeostatic imbalances observed during neurodegeneration could be associated with a GJ-independent increased membrane permeability related to HCs activity in the CNS (Retamal et al., 2007; Orellana et al., 2010; Burkovetskaya et al., 2014). In addition, increased secondary damages via cytotoxicity and inflammatory response, lead to secondary cell death and propagation of neuronal loss (O'Carroll et al., 2013; Akopian et al., 2014). This mechanism underlies a number of degenerative disorders, including retinopathies, such as glaucoma (Akopian et al., 2014, 2017), traumatic brain injury (Davidson et al., 2015b; Chen et al., 2016), stroke (Nakase et al., 2009; Orellana et al., 2014) as well as degenerative disorders of the CNS such as Alzheimer's disease (Nagy et al., 1996; Orellana et al., 2011b) and amyotrophic lateral sclerosis (ALS)-related motor neuron loss (Almad et al., 2016). These pathological conditions are characterized by reactive astrogliosis, mononuclear phagocytes activation, neuronal injury, and cell death typically linked to affected activity and regulation of main Cxs of the CNS including Cx36, Cx43, Cx30, Cx32, Cx29, and Cx47 (Decrock et al., 2015b; Belousov et al., 2017). For a specific injury and stress condition, up- or down-regulation of such proteins, likely influencing gate properties of GJs and free HCs, may contribute to both neuronal death or survival, representing the “kiss of death” and the “kiss of life,” based on which Cx is expressed and on which level (Andrade-Rozental et al., 2000). Even more, the neuronal fate is linked to the intercellular or cell-to-extracellular environment propagation of “pro-death” and “pro-survival” permeable signals (Akopian et al., 2014; Decrock et al., 2015b; Belousov et al., 2017). This complex scenario is emphasized for Cx43, one of the most abundant Cxs in the CNS and main actor in mediating glial responses to CNS injury. Many studies support the potential therapeutic efficacy of Cx43-GJ blockade on cell survival, suggesting a role of the GJs and HCs activity in increasing secondary damages (Orellana et al., 2010; Bennett et al., 2012; O'Carroll et al., 2013). Recent scientific evidence supports a pivotal role for Cx43 in different mechanisms in CNS and specifically in the microenvironment of the neurovascular unit, from the regulation of the blood brain barrier (BBB) to the modulation of integrative brain functions (i.e., learning, memory, and behavior), indicating Cx43 as an attractive target for therapeutic strategies in different brain pathologies (Salmina et al., 2014). Using a pharmacological approach we recently demonstrated a neuroprotective effect on *in vitro* neuron-like cultures exposed to hypoxic stress conditions reducing cell-to-cell and cell-to-extracellular environment communication through carbenoxolone (non-selective GJs inhibitor), ioxynil octanoato (selective Cx43-based GJs inhibitor), and Gap19 (selective Cx43-based HCs inhibitor; Vicario et al., 2017). Our results were in accordance with previous evidences which demonstrated an abnormal and progressive increase in Cx43 expression, enhancing GJs-mediated coupling, and increased HCs activity, as one of the mechanisms for astrocyte-mediated toxicity in an *in vivo* model of neurodegenerative disorder (Almad et al., 2016). The use of both GJs or HCs blockers conferred neuroprotection also to motor neurons cultured with SOD^1G93A^ astrocytes, suggesting a detrimental role of Cx43 in neurodegenerative models of ALS (Almad et al., 2016). Similar protective effects of blocking Cx43 have been described in other neurodegenerative injury including hypoxia, ischemia, Alzheimer's disease, and glaucoma (Chew et al., 2010; Wang et al., 2014; Chen et al., 2016; Giaume et al., 2017).

However, experimental results support the idea that Cx43 involvement is strictly context-dependent and related to the effects of specific phosphorylation sites in the C-terminal tail and inter-protein interaction, affecting trafficking, turnover, assembly, and gating (Cooper and Lampe, 2002; Richards et al., 2004; Yoon et al., 2010; Márquez-Rosado et al., 2012; Dunn and Lampe, 2014; Davidson et al., 2015a; Schulz et al., 2015), which prevent a generalization and stimulate further investigations on Cxs involvement in neurodegenerative and neuroprotective processes.

## Concluding remarks

Our knowledge about Cxs-mediated neuroprotection is doomed to grow quickly. The possibility to potentiate endogenous neuroprotective mechanisms represents certainly a fascinating approach for powerful therapeutic applications after CNS injury. GJs and HCs involvement in maintaining the balance of CNS microenvironment strongly stimulate research toward the development of new modulators for Cxs-based channels to be used as novel therapeutic agents against CNS disorders. A number of studies have pointed out the beneficial effect of drugs targeting Cxs-based channels, paving the way to develop complementary cell-specific approaches for the treatment of a broad range of diseases. Finally, since experimental evidences solidly demonstrate that astrocytes and Cx43 have a prominent role in neurodegenerative processes, this cell population and its molecular tools, including Cx-based structures, are more and more going to be confirmed as the indispensable guardians of neuronal activities.

## Author contributions

All authors listed have made a substantial, direct and intellectual contribution to the work, and approved it for publication.

### Conflict of interest statement

The authors declare that the research was conducted in the absence of any commercial or financial relationships that could be construed as a potential conflict of interest.
